# Human liver organoids are susceptible to *Plasmodium vivax* infection

**DOI:** 10.1186/s12936-024-05202-8

**Published:** 2024-12-05

**Authors:** Norapat Nitaramorn, Porntida Kobpornchai, Nongnat Tongkrajang, Urai Chaisri, Mallika Imwong, Kasem Kulkeaw

**Affiliations:** 1 Graduate Program in Biodesign in Medicine, Department of Parasitology, Faculty of Medicine Siriraj Hospital, Mahidol University, Bangkok, 10700 Thailand; 2 Siriraj Integrative Center for Neglected Parasitic Diseases, Faculty of Medicine Siriraj Hospital, Mahidol University, Bangkok, 10700 Thailand; 3 Siriraj-Long Read Laboratory, Faculty of Medicine Siriraj Hospital, Mahidol University, Bangkok, 10700 Thailand; 4 Department of Tropical Pathology, Faculty of Tropical Medicine, Mahidol University, Bangkok, 10400 Thailand; 5 Department of Molecular Tropical Medicine and Genetics, Faculty of Tropical Medicine, Mahidol University, Bangkok, 10400 Thailand

**Keywords:** Malaria, *Plasmodium vivax*, Hepatocyte, Liver organoid, Induced pluripotent stem cells, Disease model

## Abstract

**Background:**

The eradication of *Plasmodium vivax* malaria is complicated due to the presence of hypnozoites, the hidden dormant form of the parasite that is present in the liver. Currently available drug regimens are effective at killing hypnozoites but cause side effects and are difficult to administer. Studies testing drugs for liver-stage malaria remain rare and mainly rely on the use of cancerous or immortalized hepatic cells and primary hepatocytes.

**Methods:**

Organoids were used as platform to model liver-stage vivax malaria. Hepatic endoderm cells, endothelial progenitor cells and mesenchymal cells were generated from human induced pluripotent stem cells and self-assembled into liver organoids on top of Matrigel layer. Liver characteristic and maturity were examined through genes and proteins expression of liver markers, and liver functional tests before infected with *Plasmodium vivax* sporozoites. The infection was then verified by the detection of parasitophorous vacuole membrane proteins, Upregulated in Infectious Sporozoite 4 (UIS4), and blood-stage infection following co-culture with human reticulocytes.

**Results:**

Generated liver organoids showed upregulation of liver specific transcripts including hepatic nuclear factor 4A (*HNF4A*), alpha-fetoprotein (*AFP*), and albumin (*ALB*) which also confirmed by the protein expression. Furthermore, those organoids resembled mature hepatocytes in terms of albumin secretion, fat and glycogen storage and cytochrome activity. Following invasion of *P. vivax* sporozoites, *Pv*UIS4 was detected and the hepatic merozoites could develop into ring-stage and early trophozoites in human reticulocytes. Moreover, differential expression patterns of genes involved in lipid and cholesterol synthesis were also detected.

**Conclusions:**

Stem cell-derived liver organoids resemble mature liver cells in terms of liver functions and are susceptible to infection with *P. vivax* sporozoites, paving the way for studies on the mechanism of hypnozoite formation and testing of possible hypnozoitocidal drugs.

**Supplementary Information:**

The online version contains supplementary material available at 10.1186/s12936-024-05202-8.

## Background

The liver is a pivotal organ for the initiation of successful parasitism by *Plasmodium* spp. in humans. *Plasmodium* parasites utilize two types of host cells to expand their numbers before transmitting their progeny to a new host via sexual reproduction in a female anopheline mosquito [1]. Following hepatocyte entry, infective stage sporozoites multiply asexually to produce many merozoites that subsequently egress from hepatocytes to invade and grow in erythrocytes. A single sporozoite gives rise to more than 10,000 merozoites, increasing the parasite load in the body. Liver-stage malaria is mostly asymptomatic, while intraerythrocytic growth is of medical importance due to clinical manifestations and the risk of life-threatening conditions. Thus, many attempts have been made to discover new drugs targeting intraerythrocytic growth. Given the uncomplicated nature of erythrocyte culture systems [2], a large number of drug candidates have been approved for clinical use against erythrocyte-stage malaria [3]. However, liver-stage malaria remains a valuable target for preventing progression to clinical onset.

Compared to *P. falciparum,* the most prevalent causative agent of malaria, achieving a radical cure for the liver stage of *P. vivax* infection is more challenging. Instead of undergoing cell division, *P. vivax* sporozoites persist in the liver in a dormancy stage, known as the hypnozoite stage, and cause periodic relapses. Hence, a challenge in vivax malaria eradication is the need to identify and treat individuals who harbor *P. vivax* hypnozoites, which pose a risk of disease transmission due to subclinical chronic parasitaemia [4, 5]. Currently available drug regimens including primaquine and tafenoquine, which target hypnozoites, are not safe for patients who are deficient in glucose-6-phosphate dehydrogenase (G6PD), many of whom live in malaria-endemic areas [6, 7]. Drugs and vaccines targeting liver-stage malaria and hypnozoites have the potential to eliminate and prevent malaria. Therefore, there is an urgent need to develop disease models of liver-stage malaria [8]. To screen drugs for liver-stage malaria, studies have mainly used conventional cell culture platforms. *P. vivax* sporozoites invade and develop into merozoites in hepatic cell lines, including those derived from liver cancer [9, 10] or generated via cell immortalization [11, 12]. These hepatic cell lines grow as a monolayer on the rigid surface of plastic plates. Due to their high proliferation, overgrowth of these cell lines leads to detachment from the surface, limiting long-term culture to support hypnozoite formation [9, 10]. Although the culture of primary human hepatocytes overcomes these issues related to overgrowth, these cells lose their hepatocyte identity over time and are difficult to expand [13–16]. Thus, the use of primary human hepatocytes for drug screening remains a challenge. Notably, an attempt to culture primary human hepatocytes as a microscale monolayer with fibroblast stromal cells has been reported. This platform allows long-term culture while maintaining hepatocyte functions, but it also requires highly specialized cell imaging equipment [14, 15]. Given the batch-to-batch variation in the susceptibility of primary cells to sporozoites, prescreening of cryopreserved primary human hepatocytes is required before they can be used as a liver-stage malaria model [15]. A humanized mouse model is available and is useful for modelling *P. falciparum* and *P. vivax* malaria in the liver; however, the use of this model requires animal facilities, which limit drug screening for many compounds [17, 18]. Thus, the existing models of liver-stage malaria remain limited, leaving room for improvement.

Advances in stem cell culture and biomaterials have allowed the generation of human tissue-like models. The emergence of organoid platforms has provided alternative disease models [19]. Under optimal conditions and in a three-dimensional (3D) system, adult human stem cells can differentiate into various specialized cells that self-organize resemble the human tissue architecture; these systems are called organoids. Notably, the biological functions of organoids are comparable to those of their tissue counterparts [20, 21]. Liver organoids have been generated from fetal and adult livers and even from induced pluripotent stem cells (iPSCs) [22, 23]. Recently, fetal liver-derived organoids have become a source of mature hepatocytes and have been established as organoid lines. Two-dimensional (2D) cultures of fetal liver organoid-derived hepatocytes are permissive to *P. falciparum* invasion and development [24]. Despite an undetectable level of hepatocyte-egressing merozoites, this platform can be used to elucidate early gametocytogenesis and hypnozoite formation at single-cell resolution [24]. Moreover, the use of human iPSCs avoids ethical concerns and overcomes donor shortage issues. In this study, human iPSCs were used to generate liver organoids by mixing three main cell components essential for fetal liver budding, namely, hepatic endoderm cells, mesenchymal cells and endothelial progenitor cells [25]. The susceptibility of these iPSC-derived liver organoids to sporozoite infection were tested and examined host responses to *P. vivax* sporozoites at the transcript level.

## Methods

### Ethical approval/declaration

To generate liver organoids in this study, a human iPSC line, MUSIi001-A, established in previous study [26] were used. Human erythrocytes were obtained from the peripheral venous blood of the subjects after obtaining informed consent. Protocols related to iPSCs, blood collection and cell preparation were approved by the Human Research Protection Unit, Faculty of Medicine Siriraj Hospital, Mahidol University (COA no. Si 953/2023, 924/2566(IRB3), and 400/2567(Exempt)). *P vivax* sporozoites were used in accordance with biosafety guidelines, and the corresponding protocols were approved by the Faculty of Medicine Siriraj Hospital, Mahidol University (approval no. SI 2024–003).

### Culture of human iPSCs

Human MUSIi001-A [26] were cultured in a cell culture plate coated with 2 µg/mL Matrigel (Growth Factor Reduced; Corning, BD Bioscience). The culture medium used was Essential E8 medium (Gibco, Waltham, Massachusetts) supplemented with 100 units/mL penicillin and 100 µg/mL streptomycin (Invitrogen, Waltham, Massachusetts). The cells were passaged every 5–6 days using 0.5 mM EDTA (Invitrogen, Waltham, Massachusetts) to detach the cells from the plate and dissociate the cells into clumps. The culture medium was supplemented with 10 μM Y-27632 (StemCell Technologies, Vancouver, Canada) to prevent cell death after cell passage or thawing of liquid nitrogen-frozen cells.

### Generation of hepatic endoderm cells

To obtain hepatic endoderm cells, two sequential steps were performed. For endoderm specification, human iPSCs were cultured in RPMI medium supplemented with 100 ng/mL activin A (Peprotech, Cranbury, New Jersey) and 50 ng/mL Wnt family member 3a (Peprotech, Cranbury, New Jersey) and 1xB27 (Gibco, Waltham, Massachusetts), hereafter called RPMI/B27 medium. The cells were incubated under ambient oxygen and 5% CO_2_ for 6 days. The culture medium was replaced every other day. In the second phase, the culture medium was changed to RPMI/B27 medium containing 20 ng/mL bone morphogenetic protein 4 (Peprotech, Cranbury, New Jersey) and 10 ng/mL basic fibroblast growth factor (Gibco, Waltham, Massachusetts). The cells were cultured under ambient O_2_ and 5% CO_2_ for another 2 days.

### Generation of endothelial progenitors

To generate endothelial progenitor cells, human iPSCs were first differentiated into mesoderm cells via cultivation in DMEM/F12 supplemented with 1xB27, 1xGlutaMAX (Gibco, Waltham, Massachusetts), 8 μM CHIR99021 (Sigma‒Aldrich, St. Louis, Missouri), and 25 ng/mL BMP4 (PeproTech, Cranbury, New Jersey) for 4 days under ambient O_2_ and 5% CO_2_. Then, the culture medium was changed to endothelial induction medium, which consists of StemPro34-SFM (Gibco, Waltham, Massachusetts) supplemented with 200 ng/mL vascular endothelial growth factor (PeproTech, Cranbury, New Jersey) and 2 μM forskolin (StemCell Technologies, Vancouver, Canada), for another 4 days under the same conditions.

### Generation of septum transversum mesenchyme cells

Regarding mesenchymal cells, septum transversum mesenchyme [27] cells are derived from the mesoderm and were shown to be responsible for liver budding in a mouse model [23]. STM generation was divided into three sequential steps. In the first step, human iPSCs were cultured with DMEM/F12 culture medium containing 1xB27 (Gibco, Waltham, Massachusetts), 1xGlutaMAX, 8 μM CHIR99021 (Sigma‒Aldrich, St. Louis, Missouri), and 25 ng/mL bone morphogenetic protein 4 for 4 days. Next, the culture medium was changed to DMEM/F12 supplemented with 1xB27, 1xGlutaMAX, 2 ng/mL activin A, and 10 ng/mL platelet-derived growth factor-BB (Miltenyi Biotec, Bergisch Gladbach, Germany) at a ratio of 1:1, and the cells were cultured for an additional 2 days. In the last step, the cells were cultured in STM induction medium consisting of StemPro34-SFM, 10 ng/mL basic fibroblast growth factor, and 10 ng/mL platelet-derived growth factor-BB for 2 days. All three steps involved incubation under ambient oxygen and 5% CO_2_.

### Liver organoid formation

Liver organoids were generated following a previous study, with modifications, to obtain three different cell types on the same day. Hepatic endoderm cells, endothelial progenitors, and STM cells were detached from a well of a 24-well plate using 0.25% trypsin/EDTA (Gibco, Waltham, Massachusetts). Each type of cell was resuspended in liver organoid medium containing hepatocyte culture medium (Lonza, Basel, Switzerland) and endothelial growth medium (StemPro-34 SFM containing 50 ng/mL vascular endothelial growth factor) at a 1:1 ratio. Then, the liver organoid medium was supplemented with 20 ng/mL hepatocyte growth factor (Peprotech, Cranbury, New Jersey), 10 ng/mL oncostatin-M (Peprotech, Cranbury, New Jersey), 0.1 μM dexamethasone (Peprotech, Cranbury, New Jersey), and 2% fetal bovine serum (Gibco, Waltham, Massachusetts). Then, the hepatic endoderm cells, endothelial progenitor cells, and STM cells were mixed at a ratio of 100:70:20. Next, 190 μL of the mixed cells were seeded on top of pre-solidified Matrigel, which was prepared by mixing with liver organoid medium at a ratio of 1:1, in a well of a 96-well cell culture plate. The cells were cultured under ambient O_2_ and 5% CO_2_ in a 37 °C incubator for 13 days. The culture medium was renewed every other day. On day 21, the culture medium was changed to hepatocyte culture medium (Lonza, Basel, Switzerland), and the cells were further cultured until day 33.

### Sporozoite invasion and development

Liver organoids fully develop at 33 days post iPSC differentiation, based on the release of albumin and the expression of hepatocyte-specific transcripts. Thus, 33-day liver buds were subjected to coculture with sporozoites. Cryopreserved *P. vivax* sporozoites, obtained from the Shoklo Malaria Research Unit, were thawed by dipping them in a water bath at 37 °C for 30 s and resuspending them in hepatocyte culture medium. One hundred microlitres of sporozoite suspension at a density of 5 × 10^4^ cells/mL was added to each well of a 96-well plate and incubated at 37 °C for 6 h. After 6 h of incubation, the culture medium was removed to remove the noninvaded sporozoites, and liver organoid medium was added. The infected hepatocyte culture was maintained at 37 °C, and the medium was changed daily. On days 3 and 6 post sporozoite invasion, liver-stage parasites and exoerythrocytic forms were monitored using an indirect immunofluorescence assay and quantitative reverse-transcription PCR. To evaluate the complete development of liver-stage malaria, the release of merozoites from a hepatocyte was examined. Thus, human reticulocytes were added to each well on days 6 and day 8. After 24 h of incubation, the reticulocytes were collected and attached to glass slides by preparing a thin blood smear (Fig. 3A). Giemsa staining was performed to observe the ring-shaped trophozoites.

### Sporozoite gliding motility assay

To verify the survivability of the sporozoites after thawing, a gliding motility assay was performed following a previous report [28, 29]. After thawing the cryopreserved sporozoites and adding hepatocyte culture medium, 200 µL of sporozoite suspension was transferred to a 1.5-mL tube and centrifuged at 1200*g* at 4 °C for 3 min. Following removal of the supernatant, sporozoites were resuspended in RPMI medium containing 3% bovine serum albumin (BSA) to activate them. Resuspended sporozoites were transferred to a well of the TomoDish (Tomocube, Daejeon, Republic of Korea). The movement of sporozoites was captured using HT-2H holotomographic microscope (Tomocube, Daejeon, Republic of Korea). Time-lapse images were recorded for a single sporozoite (n = 6). Sporozoite movement speed was calculated as follows: the distance (µm) is divided by time (sec).

### Immunofluorescence staining

In the wells, liver buds were soaked in cold PBS to solubilize the Matrigel. Then, the liver organoids were washed 2–3 times to remove the solubilized Matrigel. The liver buds were fixed with 4% paraformaldehyde in PBS at room temperature for 30 min, followed by paraffin embedding and sectioning. Each liver bud section was attached to a glass slide and air-dried. For analysis of single cells, cells were attached to a glass slide via cytocentrifugation. Before immunostaining, liver bud sections or cells were fixed with 4% paraformaldehyde in PBS at room temperature for 30 min and then permeabilized with 0.25% Triton X-100 in PBS for 15 min. After washing with PBS, the samples were incubated with 1% BSA in PBS for 15 min at room temperature, followed by incubation with the following antibodies at the optimal concentrations: rabbit anti-human polyclonal HNF4 antibody (1:200, Sigma‒Aldrich), mouse anti-human monoclonal α-fetoprotein antibody (1:200, Sigma‒Aldrich), rabbit anti-human polyclonal albumin antibody (1:100, Sigma‒Aldrich), rabbit anti-human polyclonal CYP3A43 antibody (1:100, Abcam), rabbit anti-human polyclonal SR-BI antibody (1:200, Abcam) and mouse anti-human monoclonal CD81 antibody (1:100, Abcam). All antibodies were diluted in 1% BSA, applied to glass slides and incubated overnight at 4 °C. After washing with PBS, the cells were incubated with Alexa Fluor 488-conjugated goat anti-mouse IgG (Invitrogen) and Alexa Fluor 488-conjugated goat anti-rabbit IgG (Invitrogen). The glass slides were mounted with DAPI-containing antifading medium and covered with a glass coverslip. Images of the cells were acquired using a confocal microscope (ECLIPSE Ti-Cl 4 Laser Unit, Nikon).

### Quantitative reverse-transcription PCR

RNA was extracted using an RNA extraction kit (Invitrogen), and cDNA was prepared using a cDNA synthesis kit (Biotech Rabbit, Berlin, Germany). The primer sets used were obtained from previous reports [30–36] and are shown in Table 1. For PCR, Luna® Universal qPCR Master Mix (New England BioLabs, Ipswich, Massachusetts) was used at a concentration of 1 mM for forward and reverse primers (Table 1). The thermal cycling parameters were as follows: initial denaturation at 95 °C for 3 min; 30 cycles of denaturation at 95 °C for 10 s, annealing at 60 °C for 10 s, and extension at 72 °C for 10 s; and a final extension at 72 °C for 1 min. Transcripts of glyceraldehyde-3-phosphate dehydrogenase (*GAPDH*) served as internal controls for normalization of gene expression levels. The threshold cycle (CT) of each sample was used to calculate the relative expression level via the 2^−ΔΔCT^ method [37]. Gene expression data were obtained from three independent experiments, and each qRT-PCR analysis was carried out in duplicate.

**Table 1:** Sequences of the primers used for quantitative RT‒PCR in this study

Name	Forward sequence (5’–3’)	Reverse sequence (5’–3’)
Human genes		
*GAPDH*	ACTGCCACCCAGAAGACTGT	CCATGCCAGTGAGCTTCC
*AFP*	GCAGCCAAAGTGAAGAGG	TGCTGCTGCCTTTGTTG
*ALB*	AGACAAATTATGCACAGTTG	TTCCCTTCATCCCGAAGTTC
*HNF4A*	GGCAATGTGTCAGGGAGGAA	CAGGGATTTCAGGGGCACTT
*CYP3A4*	CCTRACARARACACACCCTTTG	GGTTGAAGAAGTCCTCCTAAGCT
*TAPA1*	CTGCTTTGACCACCTCAGTGCT	TGGCAGCAATGCCGATGAGGTA
*SCARB1*	GGTCCAGAACATCAGCAGGATC	GCCACATTTGCCCAGAAGTTCC
*VE-Cadherin*	GAAGCCTCTGATTGGCA AGTG	TTTTGTGACTCGGAAGAACTGGC
*PECAM1*	AAGTGGAGTCCAGCCGCATATC	ATGGAGCAGGACAGGTTCAGTC
*THY1*	GAAGGTCCTCTACTTATCCGCC	TGATGCCCTCACACTTGACCAG
*ENG*	CCACTAGCCAGGTCTCGAAG	GATGCAGGAAGACACTGCTG
*APOA1*	CCCAGTTGTCAAGGAGCTTT	TGGATGTGCTCAAAGACAGC
*APOB*	CAAACTGACCAGGACTGCCT	CCCAAAGAGACCAGTCAAGC
*FASN*	CTGGAAGGCGGGGCTCTAC	AGTGTGTGTTCCTCGGAGTG
*LSS*	TACCTGCGGCACATTGAGG	GAAGAATGTTCCGGGCTCGT
*MSMO1*	TTGGAACACCTGGCGAGTC	CCACAGCCAAGGATGCTGAA
*TM7SF2*	ACACATGACGGGTTTGGCTT	GGAAGATGTAGTAACCAGTAGCATT
*P. vivax* genes		
*Pv18S rRNA*	GAAGAAAATATTGGGATACGTAACAG	ATCGGTAGGAGGCGACGGGCG
*msp1*	GAGCTCCGAGCACACATGTA	CCATGCACAGGAGGAAAAGC

### Lipid storage

Lipids stored in cells were visualized using fat-soluble Oil Red O following the manufacturer’s instructions (Abcam). The cells were attached to a glass slide via cytocentrifugation. After air drying, the glass slide was placed in propylene glycol for 2 min and then incubated with Oil Red O solution for 6 min. The glass slide was immersed in 85% propylene glycol in distilled water for 1 min and rinsed twice with distilled water. The cells were then stained with haematoxylin for 1–2 min and rinsed thoroughly in tap water. Then, the glass slide was rinsed twice with distilled water and air-dried.

### Glycogen storage

The cells were treated with 4% paraformaldehyde in PBS and permeabilized with 0.5% Triton X-100 in PBS. Cells treated with 1 mg/mL diastase in PBS (Sigma) were used as the negative control. The cells were then incubated with periodic acid for 5 min, washed with distilled water, and incubated with freshly prepared Schiff’s solution for 15 min. Finally, the cells were rinsed, and the nuclei were stained with haematoxylin. After rinsing with water, the cells were incubated with a bluing reagent for 30 s and then incubated with Light Green Solution for 2 min. The glass slide was immersed in absolute alcohol for dehydration and air drying.

### Enzyme-linked immunosorbent assay (ELISA) for human albumin

The amount of human albumin secreted into the culture medium was assessed using the Human Albumin/Serum albumin ELISA Kit (Millipore, Burlington, Massachusetts) following the manufacturer's instructions. Hepatocyte culture medium was used as a background control. Each independent experiment was performed in triplicate.

### Reticulocyte enrichment

The O-positive (O +) human blood samples were prepared by washing with cold RPMI three times, followed by centrifugation at 1000*g* at 4 ℃ for 10 min to discard the plasma and white blood cells. Packed red blood cells were diluted with cold RPMI at 20% hematocrit and incubated at 4 ℃ for 15 min. Then, 15% Optiprep-KCl were prepared from 60% Optiprep (Axis-shield, Scotland, United Kingdom) and KCl buffer (pH 7.4) containing 115 mM KCl, 20 mM HEPES, 1 mM MgCl_2_, 1 mM NaH_2_PO_4_*H_2_O, 10 mM D-glucose, 0.5 mM EGTA, and 12 mM NaCl. A total of 4 mL of cold 20% hematocrit blood were overlayered on top of 4 mL 15% Optiprep-KCl and subsequently centrifuged at 3000*g* for 30 min at 25 ℃. Reticulocytes were collected from the interface, transferred to a 50-mL tube containing 20 mL cold RPMI, and centrifuged at 1000*g* at 4 ℃ for 5 min. Reticulocytes were washed three times with cold RPMI and resuspended with RPMI to obtain 50% hematocrit and stored at 4 ℃ for further use.

### Statistical analysis

GraphPad Prism 10.2.2 (GraphPad Software, Boston, Massachusetts) was used for statistical analysis. The data are presented as the means ± standard deviations (SD). Differences were statistically evaluated using Student’s t test. p values less than 0.05 were considered to indicate statistical significance.

## Results

### Generation of human liver organoids

**Fig. 1:**
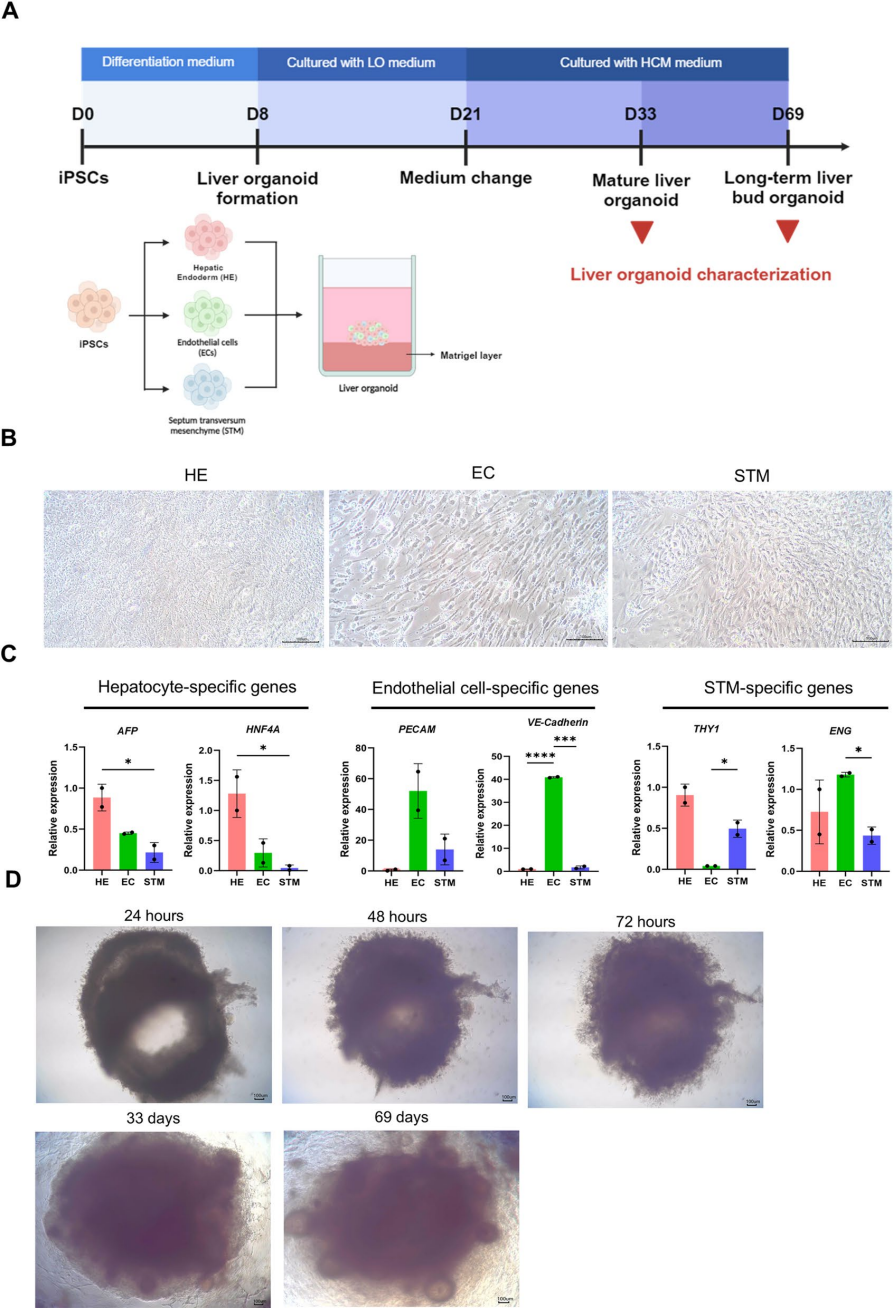
iPSC differentiation and liver organoid formation. **A** Schematic diagram showing liver organoid formation in a 3D culture system. The maturity of the liver organoids was assessed from day 33 onward. Created in BioRender. https://BioRender.com/e26k372**B** Microscopy images of iPSC-derived hepatic endoderm cells (HEs), endothelial cells (ECs), and septum transversum mesenchyme (27) cells on day 8 post-differentiation. Scale bars = 100 µm. **C** Levels of transcripts specific to each cell type, including α-fetoprotein (*AFP*) and hepatic nuclear factor 4 α (*HNF4*α) for hepatic endoderm cells, vascular endothelial cadherin (*VE-cadherin*) and platelet endothelial cell adhesion molecule (*PECAM1*) for endothelial cells, and endoglin (*ENG*) and Thy-1 cell surface antigen (*THY1*) for STM cells. The data are presented as the means ± standard deviations (SDs) (n = 2, technical replicates) and were statistically analysed using Student’s *t* test. Significant differences are indicated by *p < 0.05, ***p < 0.001, and ****p < 0.0001. **D** Microscopy images of liver organoids at 24 h, 48 h, 72 h, 33 days, and 69 days post-seeding. Scale bars = 100 µm

The protocol used to differentiate pluripotent stem cells into hepatic endoderm cells, endothelial cells and STM cells is illustrated in Fig. 1A. Microscopy images revealed the distinct morphology of the differentiated iPSCs on day 8 post cell differentiation (Fig. 1B). Compared with pre differentiated human iPSCs, hepatic endoderm cells are more cuboidal in shape. However, key morphological features of mature hepatocytes, such as round nuclei with multiple nucleoli and a high cytoplasmic-to-nuclear ratio, are still lacking [38]. Notably, the iPSC-derived endothelial cells had a spindle shape, while the morphology of the iPSC-derived mesenchymal cells was similar to that of fibroblasts, which have an elongated shape. The cells were validated by examining the expression of transcripts specific to each cell type (Fig. 1C). The hepatic endoderm cells expressed α-fetoprotein (*AFP*) and hepatic nuclear factor 4α (*HNF4α*) transcripts at higher levels than endothelial cells and STM cells. Compared with endoderm cells and STM cells, endothelial cells have more than 40- and 200-fold greater expressions of the genes encoding vascular endothelial cadherin (*VE-cadherin*) and platelet endothelial cell adhesion molecule 1 (*PECAM1*), respectively. However, STM had endoglin (*ENG*) transcripts at a level lower than the other cell types. The level of Thy-1 cell surface antigen (*THY1*) transcripts was the lowest in endothelial cells, but highly expressed by STM (Fig. 1C). Twenty-four hours after plating cells on top of the Matrigel, the cells were clumped and rounded into a translucent hollow shape (upper left, Fig. 1D). Later, after 48–72 h of culture, the hollow area decreased; the hollow area ultimately filled with cells and became less translucent. From day 21 onward, the liver organoid medium was replaced with hepatocyte culture medium to induce the maturation of hepatocytes. At 33 days post-cell differentiation, the liver organoid was nontranslucent and had a well-defined edge, with a diameter of 2.2 mm. The edge of the liver organoid became more diffuse as the cells spread over the Matrigel (lower panels, Fig. 1D). Notably, the Matrigel lost its ability to solidify and became liquid on days 60–69 after liver organoid formation.

### Characterization of the liver organoids

**Fig. 2:**
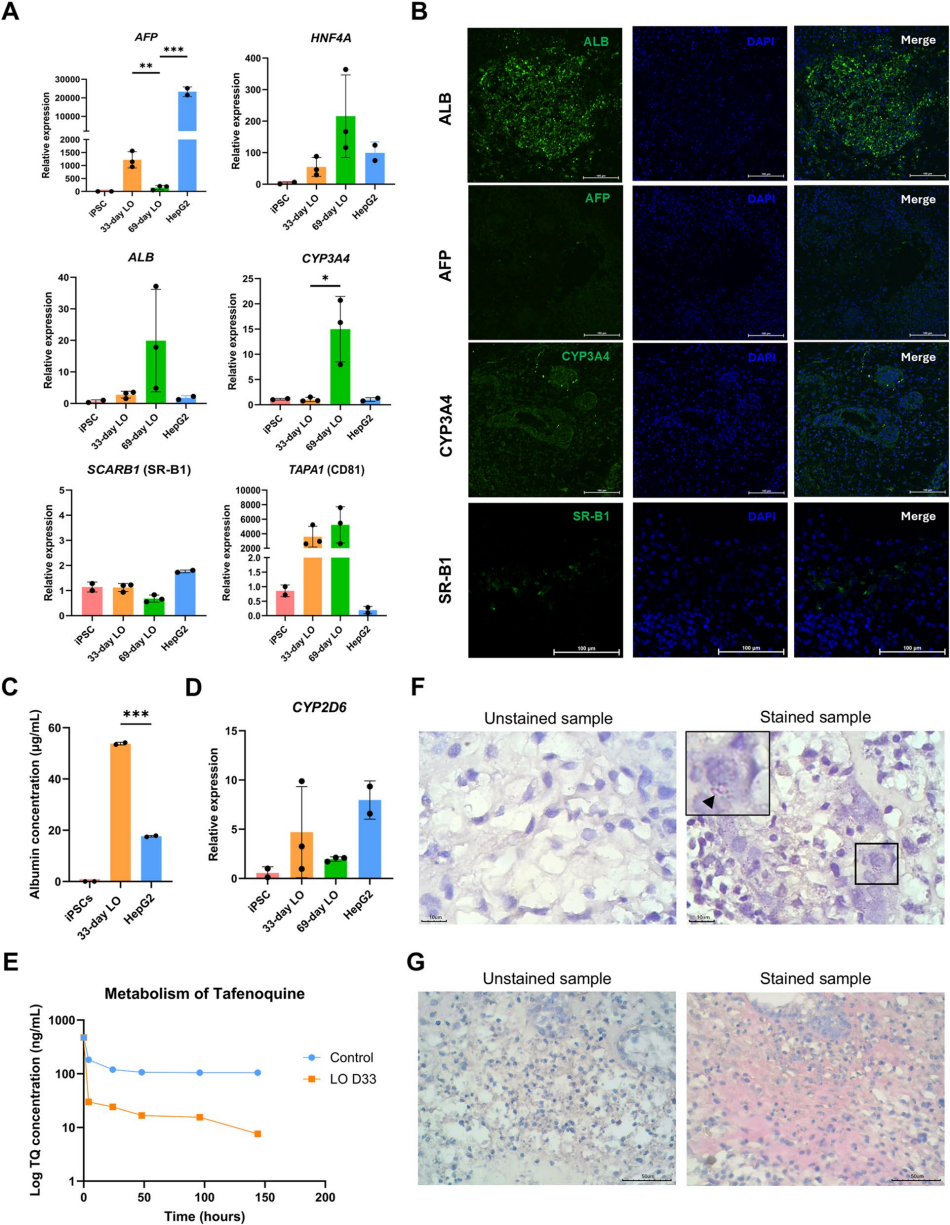
Liver organoid characterization. **A** Gene expression profile of liver organoids (LOs) on day 33 and day 69 showing the mature hepatocyte markers α-fetoprotein (*AFP*), albumin (*ALB*), hepatic nuclear factor 4 α (*HNF4α*), and cytochrome P3A4 (*CYP3A4*), as well as host cell receptors for *Plasmodium* sporozoites (*CD81*, *SR-BI*). Relative expression levels were calculated using the 2^−ΔΔCT^ method. The data are presented as the means ± SDs (n = 2, technical replicates of the iPSCs and HepG2 cells; n = 3, biological replicates of the LOs) and were statistically analysed using Student’s *t* test. Significant differences are indicated by *p < 0.05, **p < 0.01, and ***p < 0.001. **B** Confocal microscopic observation of AFP, ALB, CYP3A4, and SR-BI. Scale bars = 100 µm. **C** Total amount of albumin (µg/mL) secreted into the culture medium of day 33 liver organoids compared with iPSCs and HepGe2 cells (***p < 0.001, Student’s t-test). **D** Gene expression of the cytochrome P2D6 transcript (*CYP2D6*). The data are presented as the means ± SDs (n = 2, technical replicates of the iPSCs and HepG2 cells; n = 3, biological replicates of the 33-day LOs and 69-day LOs). **E** Drug metabolism of Tafenoquine in liver organoids. The amount of tafenoquine (ng/mL) in the culture medium was measured at different time points, including 4, 24, 48, 96, and 144 h after addition of the drug. The control group included drugs without liver organoids. **F, G** Lipid and glycogen staining of day 33 liver organoids. Representative images are shown. Scale bars = 10 µm for lipid staining and 50 µm for glycogen staining

To characterize the maturity of hepatocytes in the liver organoids, the gene expression of *AFP* and albumin (*ALB*) was first examined to determine the presence of hepatoblasts and hepatocytes, respectively. The human hepatocarcinoma HepG2 cell line was used for comparing gene expression with liver organoids. The expression levels of genes in human iPSCs were set as the baseline (set as 1, Fig. 2A). The hepatocyte-specific transcription factor *HNF4α* was upregulated in liver organoids and HepG2 cells but not in iPSCs, suggesting hepatic lineage differentiation. *HNF4a* transcript levels were higher in the 69-day liver organoids than in the 33-day liver organoids. Expression of the *AFP* transcript is detected in hepatoblasts and is highly upregulated in HepG2 cells. The 33-day liver organoids had higher *AFP* transcript levels than did the 69-day liver organoids but still had much lower *AFP* transcript levels than did the HepG2 cells. In contrast, the 33-day liver organoids had *ALB* transcript levels equivalent to those of the HepG2 cells. *ALB* transcript levels increased up to tenfold in the 69-day liver organoids. The cytochrome P450 *CYP3A4* is also expressed in mature hepatocytes. Thus, the maturity of the hepatocytes in the 69-day liver organoids were confirmed. The expression level of the *CYP3A4* transcript was highest in the 69-day liver organoids. Considering the levels of the *ALB* and *CYP3A4* transcripts, a longer period of liver organoid culture allows more mature hepatocytes to form in the liver organoids. To ensure the applicability of liver organoids for modeling liver-stage malaria, the expression of known sporozoite receptors, namely, scavenger receptor class B type 1 (*SR-BI*) and *CD81* were examined. The levels of the *SR-B1* and *CD81* transcripts were comparable between the 33- and 69-day-old liver organoids. However, the liver organoids expressed *SR-B1* transcripts at lower levels than HepG2 cells did.

Considering the gene expression of *ALB* and *CYB3A4* together with the stability of Matrigel, 33-day-old liver organoids were selected for sporozoite invasion. To confirm gene expression, immunofluorescence of liver organoid sections were preformed to detect the protein expression of AFP, ALB, CYP3A4 and SR-B1, which are entry receptors for *P. vivax* sporozoites [39], in 33-day-old liver organoids (Fig. 2B). To distinguish true positive fluorescence signals, the fluorescence background generated from liver organoid sections probed with secondary antibodies was used for comparison. The optimal conditions for antibody-antigen binding were confirmed using HepG2 cells as a positive control (Additional file 1: Figure S1A). Different patterns of fluorescence were observed. The AFP-positive cells were scattered, while the ALB-positive cells were clustered. The distribution of CYP3A4-positive cells was similar to that of AFP-positive cells. Notably, cells expressing SR-BI were detected at a specific location. After 33 days, the liver organoids secreted albumin into the culture medium (Fig. 2C and Additional file 1: Figure S1B) and expressed the *CYP2D6* transcript, which is required for antimalarial drug metabolism (Fig. 2D). Consequently, the day 33 LOs were able to metabolize tafenoquine, an effective drug for liver-stage malaria and hypnozoites (Fig. 2E). Moreover, the 33-day-old liver organoids stored lipids and glycogen. Compared to the unstained control (only H&E stain), lipids appeared as droplets in the cytoplasm (red, Fig. 2F), while the glycogen stored in the cells in the cytoplasm is indicated by a magenta colour (Fig. 2G). When comparing the secreted albumin in the culture medium at different time points starting from the 33-day LO onward, the albumin level gradually decreased over time (Additional file 1: Figure S1B). Thus, the 33-day LOs were selected for sporozoite infection.

### Intrahepatic development of *P. vivax* in liver organoids

**Fig. 3:**
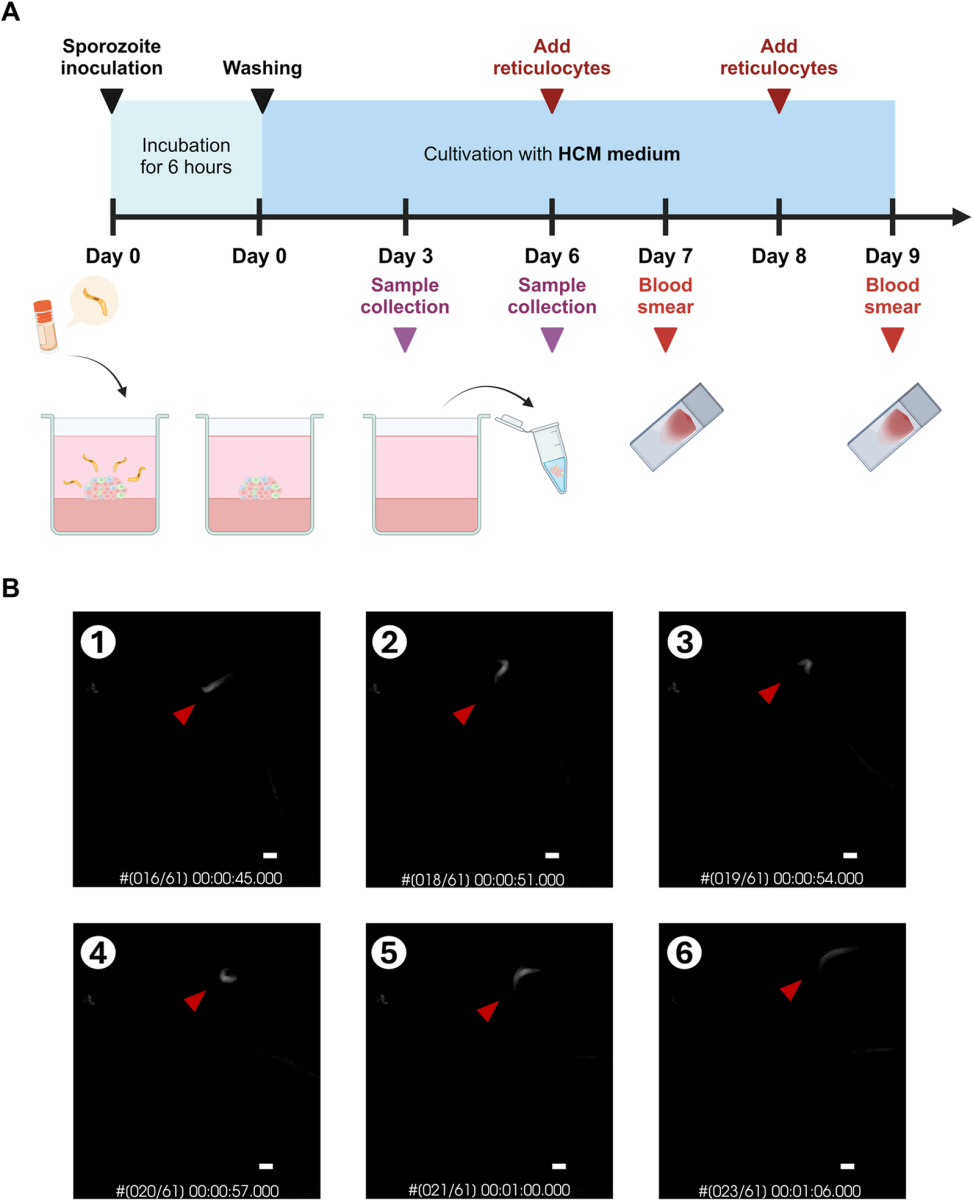
*P. vivax* infection in liver organoids. **A** Schematic diagram showing *P. vivax* infection in liver organoids. Liver organoids collected on day 33 were used for infection. Created in BioRender. https://BioRender.com/r42z460**B** Sporozoite gliding assay showed the time lapse images of sporozoite captured by holotomography techniques. Red pointers indicated same sporozoite in different positions at different time points. Scale bar = 2 µm

**Fig. 4:**
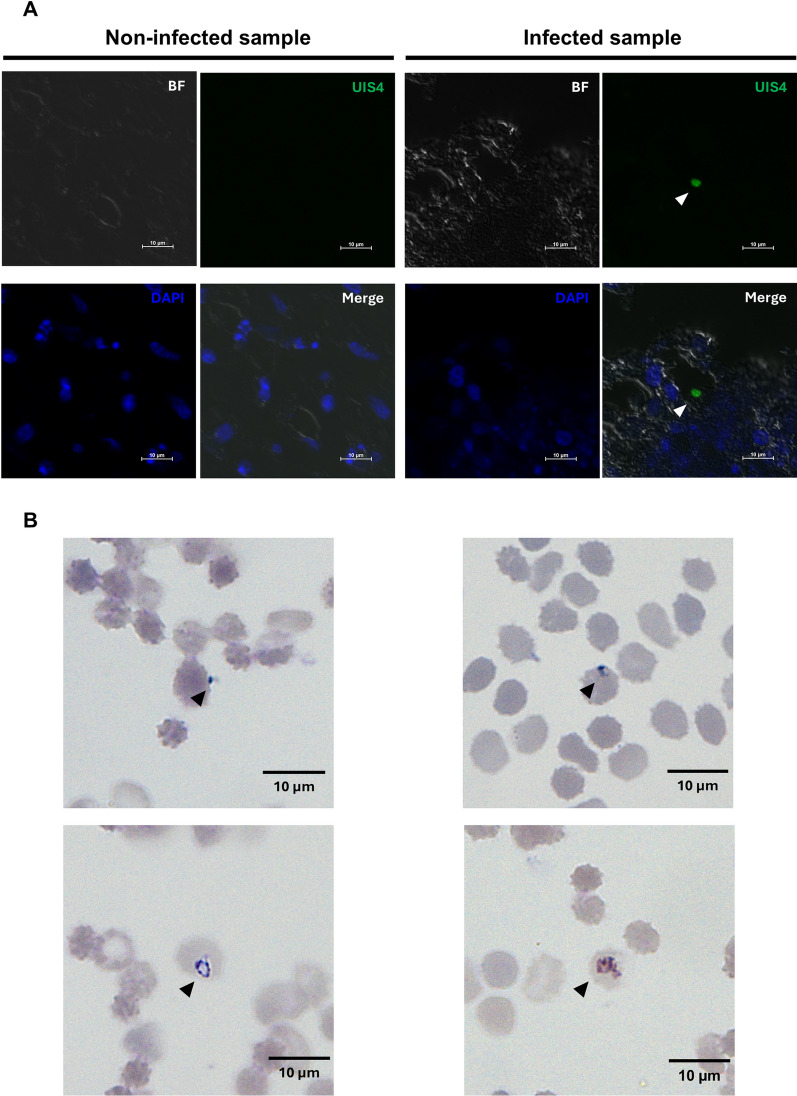
Liver-stage and blood-stage infection of *P. vivax* in liver organoids. **A** Confocal microscopic observation of *P. vivax* UIS4 in the liver organoids. Sporozoites were inoculated with the LOs. At day 6 post-inoculations, the LOs were collected and subjected to prepare a tissue section for immunofluorescence. Under the confocal images, nuclei were stained with DAPI (blue), while *P. vivax* UIS4 was detected using anti-*Pv*UIS4 (green). Bright field (BF) images show the edge of the cell layer. Scale bar = 10 µm. **B** Representative microscopic images of *Plasmodium*-parasitized reticulocytes. Human reticulocytes were added into the LOs that were inoculated with *P. vivax* sporozoite for 6 days. At 24 h post reticulocyte adding, thin blood smears were prepared and stained with Giemsa. Scale bar = 10 µm

According to previous studies, it takes 9–11 days for sporozoites to proliferate and egress from hepatic cells [15]. The protocol for the infection experiment is shown in Fig. 3A; sporozoites were inoculated with day 33 liver organoids for 6 h before washing. Infected liver organoid samples were collected on days 3 and 6 post-inoculation. Cryopreserved *P. vivax* sporozoites were used for invasion and development assays. Under a holotomographic microscope, more than 70–80% of the sporozoites exhibited gliding motility (Additional file 6: Movie), and the sporozoite time-lapse images were captured following the single sporozoite movement (Fig. 3B). The sporozoite movement speed was 0.49 ± 0.099 µm/sec (n = 6 sporozoites). To evaluate the parasite burden in association with host cell susceptibility, quantitative RT‒PCR were used to measure the levels of *Plasmodium*-specific transcripts in individual hepatocytes. *Plasmodium vivax 18 s rRNA* transcript could not be detected in the LOs at days 3 and 6 post-inoculation (Additional file 2: Fig. S2A). Then, target-specific preamplification was performed following the previous report [40] to increase the amount of cDNA template. The pool of primer set specific to human transcripts (*GAPDH* and *ALB*) and *P. vivax* (*18S rRNA* and *MSP1*). Nevertheless, only human transcripts were detected at a higher amount (lower threshold cycle, Additional file 2: Figure S2B), indicating success in preamplification. To verify the sporozoite infection at the protein level, immunofluorescence was performed using an antibody specific to *P. vivax* Upregulated in Infectious Sporozoite 4 (*Pv*UIS4), which is a parasitophorous vacuole membrane protein. The fluorescence signal of *Pv*UIS4 was bright and exhibited as a round from proximal to the nucleus of the cells in the LOs inoculated with sporozoites for 6 days. There was no fluorescence signal of *Pv*UIS4 detected in the non-infected LOs (Fig. 4A and Additional file 3: Fig. S3). Next, the egression and infectivity of the intrahepatic merozoite was examined by adding human reticulocytes isolated from peripheral venous blood on day 6 and day 8-post sporozoite inoculation (Fig. 3A). To rule out the possibility that reticulocytes are not susceptible, the expression of the transferrin receptor CD71, a receptor known to be essential for merozoite invasion, were examined. The human reticulocytes expressed CD71 and glycophorin A (Additional file 5: Fig. S5). Hence, human reticulocytes were added into the wells of the LOs after sporozoite infection for 6 and 8 days. Following 24 h of co-incubation, the Giemsa-stained blood smears were prepared and subjected to microscopic observation. Trophozoites with ring and amoeboid forms were observed in the blood smear prepared from the LOs infected with sporozoites for 6 days. Among 7000 examined human red blood cells, parasitaemia was 0.11%. In contrast, blood-stage malaria was undetectable when the LOs were inoculated with sporozoites for 8 days (Fig. 4B). Thus, *P. vivax* sporozoites invade and develop into erythrocyte-infectious merozoites in liver organoids but at a very low parasite burden.

### Changes in lipogenesis in the liver organoids after *P. vivax* invasion

**Fig. 5:**
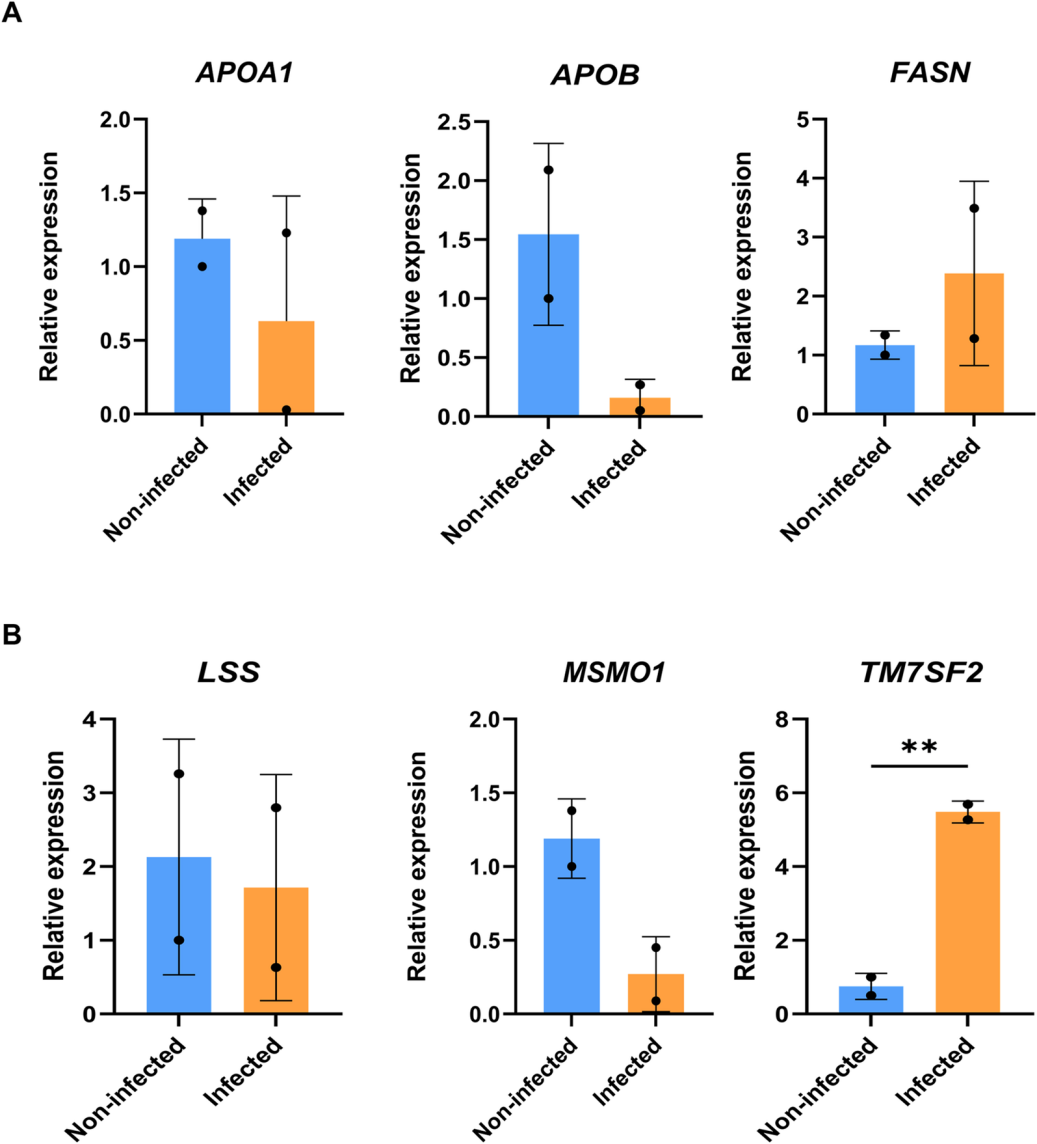
Gene expression of lipogenesis-related genes in infected and noninfected liver organoids. **A** Gene expression of transcripts related to lipid metabolism, including apolipoprotein A1 (*APOA1*), apolipoprotein B (*APOB*), and fatty acid synthase (*FASN*). **B** Gene expression of transcripts related to cholesterol metabolism, including lanosterol synthase (*LSS*), methyl sterol monooxygenase 1 (*MSMO1*) and transmembrane 7 superfamily 2 (*TM7SF2*). Human *GAPDH* was used as an endogenous control. Relative expression levels were calculated using the 2^−ΔΔCT^ method. The data are presented as the means ± SDs (n = 2, biological replicate) and were statistically analysed using Student’s *t* test. Significant differences are indicated by **p < 0.01

To demonstrate the use of liver organoids as a model for investigating host responses, the expression of genes whose expression has been reported to be altered following *Plasmodium* infection, were examined. A study performed in fetal liver organoids revealed that the lipogenesis pathway was upregulated in response to sporozoite invasion [24]. Thus, the examination of the expression levels of the following transcripts were performed: (1) genes encoding lipid transporters in blood, such as apolipoprotein A1 (*APOA1*) and apolipoprotein B (*APOB*); (2) genes encoding enzymes involved in fatty acid synthesis, such as fatty acid synthases (*FASN*); and (3) genes involved in cholesterol synthesis, including lanosterol synthase (*LSS*), methyl sterol monooxygenase 1 (*MSMO1*) and transmembrane 7 superfamily 2 (*TM7SF2*), according to protein‒protein interactions (Additional file 4: Fig. S4). The day 33 liver organoids were used as a noninfected control sample. At 9 days after sporozoite inoculation, the transcript level of *TM7SF2* significantly increased (by fivefold) (*p* < 0.01); the transcript level of *FASN* also increased, but the difference was not significant (*p* = 0.39). In contrast, the transcript levels of *APOA1, APOB, MSMO1* and *LSS* tend to decrease but did not exhibit a significant difference (*p* = 0.46, 0.13, 0.07, and 0.81, respectively) (Fig. 5A, B).

## Discussion

The presence of *P. vivax* parasitophorous vacuole protein and the egress of merozoites are evidence of sporozoite development in human liver organoids, emphasizing the susceptibility of human iPSC-derived liver organoids to *P. vivax*. The transcription changes observed in the liver organoids in response to sporozoite development recapitulate those observed in human fetal liver organoids [24].

Here, the method deployed for the liver organoid generation is optimal. There are two ways to generate liver organoids using Matrigel as a scaffold: embedding or topping. Matrigel embedding reportedly allows liver organoid formation [21, 31, 41]. However, the gel-based scaffold forms a compact structure, possibly preventing the penetration of sporozoite and traps the merozoites released from hepatocytes in the liver organoids. Takebe et al*.* [22] generated liver bud organoid by adding a cell mixture on top of the Matrigel. Thus, it allows direct exposure of the organoid to the sporozoites in a culture medium as well as the release of merozoites into the culture medium. By using the on-top gel method, the *P. vivax* sporozoites could invade and release merozoites infectious to human reticulocytes, implying the optimal method for modelling liver-stage malaria.

Moreover, prolonged culture of the liver organoid allows maturity of hepatocytes, to which the *P. vivax* sporozoites could productively infect. During the culture on days 8 to 21, the liver organoid was maintained in a liver organoid (LO) medium containing the HCM, endothelial cell growth-promoting factor (VEGF), and hepatocyte differentiation-inducing factors (HGF, OSM, Dexamethasone). Following the previously published protocol, the HCM medium could maintain albumin and urea secretion, and cytochrome activity compared with other culture conditions [42]. Therefore, the LO medium was replaced with the HCM from day 21 onward. The level of albumin secretion remains detectable from days 33 to 45 before dropping on days 51 to 63, suggesting the maintenance of hepatocyte phenotype (Additional file 1: Figure S1B). Together, the protocol of human liver organoid generation is applicable for modeling liver-stage malaria.

Studies of liver-stage malaria models unveil changes in host lipid homeostasis. Spatial transcriptome analysis shows an alteration in the lipid metabolism of hepatocytes proximal to the infection site in rodent malaria model [43]. *Plasmodium berghei* parasites acquire lipids and cholesterol from the hepatocytes located proximal to the infected one. In the human context, Yang et al*.* [24] reported an increase in transcripts encoding proteins functioning in lipogenesis after sporozoite invasion. Similar to this study, the *TM7SF2* gene functioning in cholesterol synthesis is upregulated. In contrast, lipid-transporting afpolipoprotein A1 (*APOA1*) and cholesterol-synthesizing methyl sterol monooxygenase 1 (*MSMO1*) down-regulated. The discrepancy is possibly caused by the difference in the deployed models and *Plasmodium* species. Yang et al*.* [24] generated liver organoids from primary hepatocytes obtained from a fetal liver, whereas our study combined hepatic endoderm with endothelial cells and mesenchymal to generate the liver organoid.

A limitation of 3D organoid models includes difficulty in detecting merozoites egressing from the individual hepatocytes. Notably, the gel scaffold or compacted structure likely traps the merozoites inside the liver organoids [31]. Formation of the liver organoids on top of Matrigel layer can address this problem. Although the initial attempt to detect the merozoite failed, shortening the period after sporozoite inoculation enables us to detect the blood-stage parasites. Unlike *P. falciparum*, monitoring *P. vivax* development is more complicated because the parasites require reticulocytes to develop. To ensure the quality of the reticulocytes, the expression of the transferrin receptor CD71, a known receptor of *P. vivax* merozoites was examined [44]. Thus, the complete liver schizogony of *P. vivax* was demonstrated based on success in reticulocyte invasion.

Another challenge is the difficulty of imaging cells in liver organoids that are organized in a 3D manner. The cells couldn’t be observed under an inverted light microscope. Thus, liver organoid sectioning is necessary. Unlike 2D culture, high-resolution cell imaging allows assessment of infection rates, parasite growth and drug concentrations. In the culture of human primary hepatocytes surrounded by mouse fibroblasts, sporozoite invasion and proliferation could be monitored in a well of a 384-well plate in real time [15]. In addition, accurate determination of the infection rate is hampered by the need for immunodetection of representative liver organoid sections. To address this limitation, the rate of sporozoite infection of hepatocytes can be calculated based on the level of a parasite-specific transcript relative to that of *ALB*-expressing hepatocytes. However, the infection rate proposed here might be overestimated due to the downregulation of human *ALB* transcripts in primary human hepatocytes cocultured with mouse fibroblasts [45]. Thus, the use of *ALB* transcripts to calculate infectivity needs to be done carefully. Nevertheless, a low infection rate may overcome this limitation because only a small fraction of hepatocytes is infected with sporozoites. Since the rate of sporozoite infection reportedly doesn’t correlate with gliding activity [46]. The gliding motility assay confirms that a high proportion of sporozoites remain active and survive after thawing. Hence, a low infection rate was unlikely to result from the use of the cryopreserved sporozoites.

Human iPSC-derived 3D disease models are expensive and time consuming. A multistep protocol is required to prepare three distinct cell types, which must be verified before liver organoid formation. Considering the possibility of long-term culture, liver organoids may serve as a model for the reactivation of long-term persistent hypnozoites. Nevertheless, another drawback is the inconsistency in the quality of Matrigel. The protein content varied among batches [47]. Advances in materials science have led to the replacement of Matrigel with other scaffolds containing synthetic peptides cross-linked as reticular structures to support cell adhesion and organization [48]. A Matrigel-free platform recently allowed the formation of liver organoids from iPSCs. Thus, this platform may reduce the physical barrier of gel-based systems during sporozoite invasion [49]. Further improvements in the use of scaffold-dependent and scaffold-free systems will improve the similarity of liver organoids to the human liver [50].

## Conclusion

These liver organoids resemble mature hepatocytes in terms of albumin secretion, fat and glycogen storage, and cytochrome activity. *Plasmodium vivax* sporozoites invade the liver organoid and completely develop into liver-stage schizonts followed by the release of the merozoites that are infectious to human red blood cells. Changes in the transcript levels of genes involved in cholesterol synthesis were also observed. Thus, stem cell-derived liver organoids are susceptible to infection with *P. vivax* sporozoites, paving the way for studies on the mechanism of hypnozoite formation and testing of possible hypnozoitocidal drugs.

## Supplementary Information

**Additional file 1: **Figure S1. Liver organoid formation: (A) Confocal microscopy analysis of AFP, ALB, CYP3A4, and SR-BI in HepG2 cells. Scale bar = 100 µm. (**B**) Total amount of albumin (ng/mL) secreted into the culture medium of liver organoids on days 33, 39, 45, 51, 57, and 63. The data are presented as the means ± SDs (n = 2, biological replicates).

**Additional file 2: **Figure S2. Quantitative reverse transcription-PCR (qRT-PCR) with and without preamplification of cDNA. Following RNA extraction and DNase treatment to remove gDNA, cDNA was synthesized and subjected to PCR for amplifying *Plasmodium 18 s rRNA*, *MSP1*, human *GAPDH* and human albumin (*ALB*) transcript. (**A**) Amplification curves and melt peaks of amplicons obtained from qRT-PCR without preamplification of cDNA template. In the upper panels, the amplification curves show the thermal cycle numbers and the relative fluorescence unit of SYBR green on the x- and y-axis. *P. vivax*-infected liver organoid (LO) and non-infected LOs were subjected to qRT-PCR. Technical duplicate wells were performed. In the lower panels, the melt peaks indicate the melting temperature (x-axis) of the amplicons. The melting temperature is dependent on the DNA sequence. Thus, each melt peak is relatively specific to a given amplicon. (**B**) Amplification curves and melt peaks of amplicons obtained from qRT-PCR with preamplification of cDNA template. Following preamplification, the cDNA template of human *GAPDH* and *ALB* gene increased and could be detected at the early thermal cycle of amplification (a lower threshold cycle or Ct value). Preamplification of cDNA template did not cause non-specific amplification because the melting temperature of the amplicons remains unchanged (Lover panels in A and B). However, *Plasmodium 18 s rRNA* and *msp1* couldn’t be detected regardless of preamplification. Thus, the amount of *P. vivax* mRNA is relatively low in the LOs inoculated with sporozoites. gDNA of *P. vivax* was used as a positive control template for amplifying gene encoding *Plasmodium 18 s rRNA* and *msp1*.

**Additional file 3: **Figure S3. Liver-stage infection of *P. vivax* in liver organoid. (A) Confocal microscopic observation of *P. vivax* UIS4, a protein located in parasitophorous vacuole membrane, in liver organoids. Under confocal images, nuclei were stained with DAPI (blue), while *P. vivax* UIS4 was detected using anti-*Pv*UIS4 (green). Bright field (BF) images show the edge of the cell layer. Scale bar = 10 µm.

**Additional file 4: **Figure S4. Protein‒protein interactions related to lipid and cholesterol metabolism in the liver. (A) Protein‒protein interaction (PPI) networks were constructed with Metascape. The different clusters are labeled with different colours.

**Additional file 5: **Figure S5. Enrichment of human reticulocytes and surface marker identification. (A) Human reticulocytes after enrichment with 15% OptiPrep-KCl and staining with methylene blue dye. The reticulocytes presented precipitated blue RNA granules in the red cells (arrowhead). The percentage of reticulocytes was calculated under a light microscope for a minimum of 5,000 erythrocytes. (**B**) Dot plot of a representative flow cytometry analysis showing the reticulocytes before and after enrichment. The red blood cells (RBCs) were gated according to forward scatter height (FSC-H) and side scatter height (SSC-H). The reticulocytes were defined as CD71-positive and CD235a-positive cells.

**Additional file 6: **Movie. Time-lapse video of *P. vivax* sporozoite movement. Images were captured every 3 s using a holotomographic microscope (Tomocube). A total of six sporozoites were recorded, and only a representative sporozoite is shown in this video. Scale bar = 2 µm.

## Data Availability

The datasets used and/ or analyzed during the current study are available from the corresponding author on reasonable request.

## Abbreviations

G6PD: Glucose-6-phosphate dehydrogenase
2D/ 3D: Two-dimensional/ Three-dimensional
iPSCs: Induced pluripotent stem cells
STM: Septum transversum mesenchyme
BSA: Bovine serum albumin
*GAPDH*: Glyceraldehyde-3-phosphate dehydrogenase
SD: Standard deviation
*AFP*: Alpha-fetoprotein
*HNF4A*: Hepatic nuclear factor 4a
*VE-cadherin*: Vascular endothelial cadherin
*PECAM1*: Platelet endothelial cell adhesion molecule 1
*THY1*: Thy-1 cell surface antigen
*ENG*: Endoglin
*ALB*: Albumin
*CYP3A4*: Cytochrome P450 3A4
*SR-B1*: Scavenger receptor class B type 1
*APOA1*: Apolipoprotein A1
*APOB*: Apolipoprotein B
*FASN*: Fatty acid synthase
*LSS*: Lanosterol synthase
*MSMO1*: Methyl sterol monooxygenase 1
*TM7SF2*: Transmembrane 7 superfamily 2
*MSP1*: Merozoite surface protein 1
