# Supplementary material for: Human liver organoids are susceptible to Plasmodium vivax infection
Source: Malar J. 2024 Dec 5;23:368. doi: 10.1186/s12936-024-05202-8 (PMC11622667; doi:10.1186/s12936-024-05202-8)
Supplement: Supplementary file 1 — Additional file 1: Figure S1. Liver organoid formation: (A) Confocal microscopy analysis of AFP, ALB, CYP3A4, and SR-BI in HepG2 cells. Scale bar = 100 µm. (B) Total amount of albumin (ng/mL) secreted into the culture medium of liver organoids on days 33, 39, 45, 51, 57, and 63. The data are presented as the means ± SDs (n = 2, biological replicates). [file 12936_2024_5202_MOESM1_ESM.pdf]

Additional file 1

A

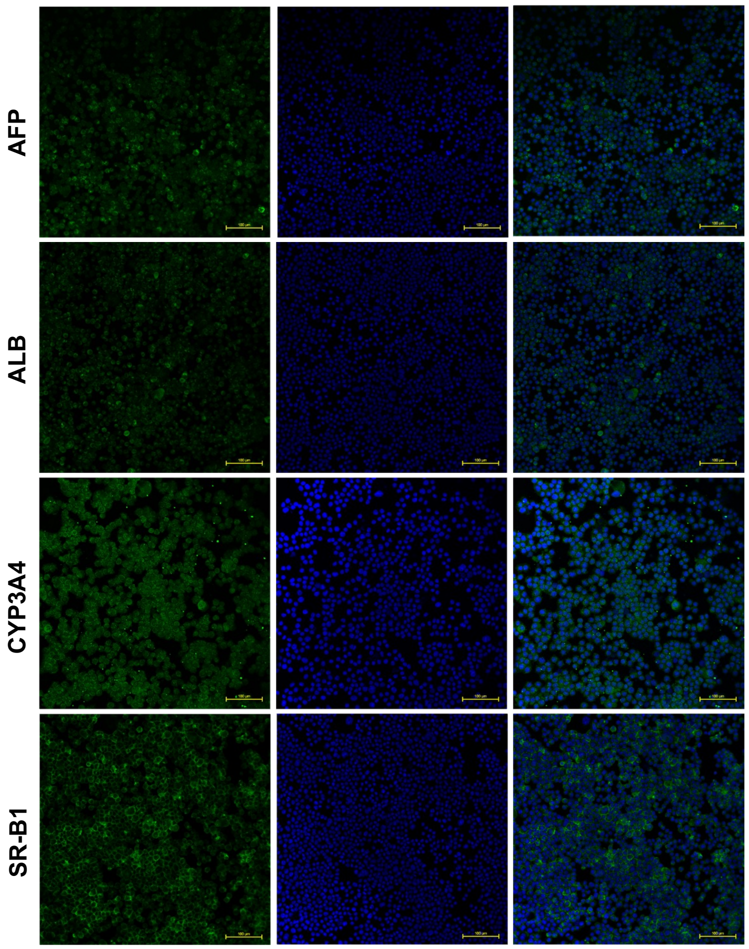

B

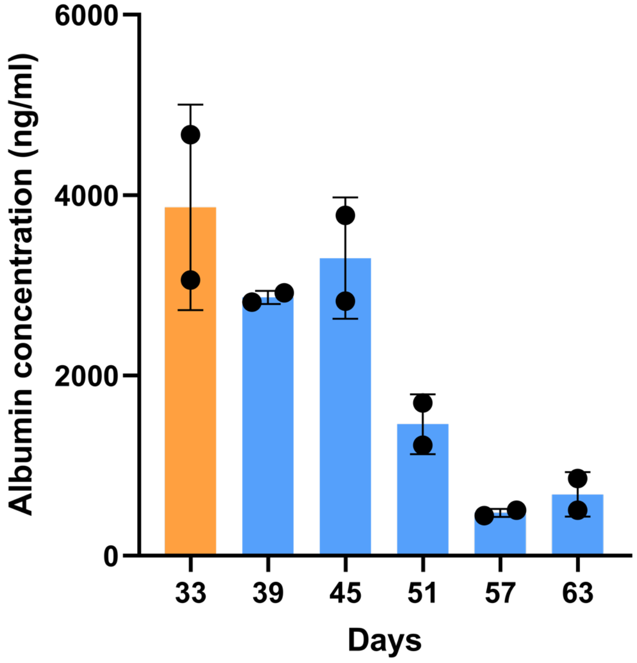

Figure S1. Liver organoid formation: (**A**) Confocal microscopy analysis of AFP, ALB, CYP3A4, and SR-BI in HepG2 cells. Scale bar = 100 μm. (**B**) Total amount of albumin (ng/mL) secreted into the culture medium of liver organoids on days 33, 39, 45, 51, 57, and 63. The data are presented as the means ± SDs (n = 2, biological replicates).
