# Supplementary material for: Human liver organoids are susceptible to Plasmodium vivax infection
Source: Malar J. 2024 Dec 5;23:368. doi: 10.1186/s12936-024-05202-8 (PMC11622667; doi:10.1186/s12936-024-05202-8)
Supplement: Supplementary file 2 — Additional file 2: Figure S2. Quantitative reverse transcription-PCR (qRT-PCR) with and without preamplification of cDNA. Following RNA extraction and DNase treatment to remove gDNA, cDNA was synthesized and subjected to PCR for amplifying Plasmodium 18 s rRNA, MSP1, human GAPDH and human albumin (ALB) transcript. (A) Amplification curves and melt peaks of amplicons obtained from qRT-PCR without preamplification of cDNA template. In the upper panels, the amplification curves show the thermal cycle numbers and the relative fluorescence unit of SYBR green on the x- and y-axis. P. vivax-infected liver organoid (LO) and non-infected LOs were subjected to qRT-PCR. Technical duplicate wells were performed. In the lower panels, the melt peaks indicate the melting temperature (x-axis) of the amplicons. The melting temperature is dependent on the DNA sequence. Thus, each melt peak is relatively specific to a given amplicon. (B) Amplification curves and melt peaks of amplicons obtained from qRT-PCR with preamplification of cDNA template. Following preamplification, the cDNA template of human GAPDH and ALB gene increased and could be detected at the early thermal cycle of amplification (a lower threshold cycle or Ct value). Preamplification of cDNA template did not cause non-specific amplification because the melting temperature of the amplicons remains unchanged (Lover panels in A and B). However, Plasmodium 18 s rRNA and msp1 couldn’t be detected regardless of preamplification. Thus, the amount of P. vivax mRNA is relatively low in the LOs inoculated with sporozoites. gDNA of P. vivax was used as a positive control template for amplifying gene encoding Plasmodium 18 s rRNA and msp1. [file 12936_2024_5202_MOESM2_ESM.pdf]

Additional file 2

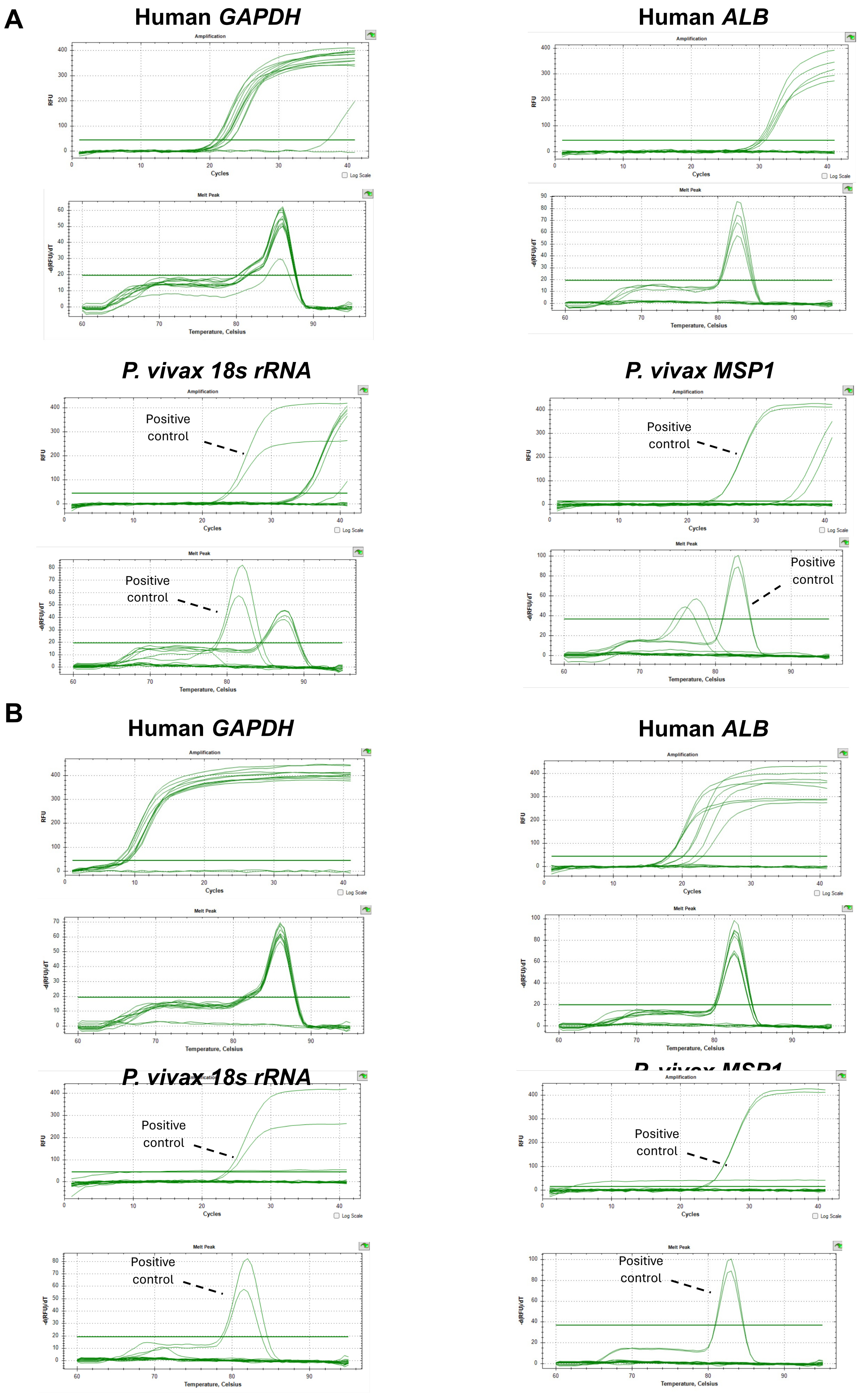

Figure S2. Quantitative reverse transcription-PCR (qRT-PCR) with and without preamplification of cDNA. Following RNA extraction and DNase treatment to remove gDNA, cDNA was synthesized and subjected to PCR for amplifying *Plasmodium 18s rRNA*, *MSP1*, human *GAPDH* and human albumin (*ALB*) transcript. (**A**) Amplification curves and melt peaks of amplicons obtained from qRT-PCR without preamplification of cDNA template. In the upper panels, the amplification curves show the thermal cycle numbers and the relative fluorescence unit of SYBR green on the x- and y-axis. *P. vivax*-infected liver organoid (LO) and non-infected LOs were subjected to qRT-PCR. Technical duplicate wells were performed. In the lower panels, the melt peaks indicate the melting temperature (x-axis) of the amplicons. The melting temperature is dependent on the DNA sequence. Thus, each melt peak is relatively specific to a given amplicon. (**B**) Amplification curves and melt peaks of amplicons obtained from qRT-PCR with preamplification of cDNA template. Following preamplification, the cDNA template of human *GAPDH* and *ALB* gene increased and could be detected at the early thermal cycle of amplification (a lower threshold cycle or Ct value). Preamplification of cDNA template did not cause non-specific amplification because the melting temperature of the amplicons remains unchanged (Lover panels in A and B). However, we failed to detect *Plasmodium 18s rRNA* and *MSP1* regardless of preamplification. Thus, the amount of *P. vivax* mRNA is relatively low in the LOs inoculated with sporozoites. gDNA of *P. vivax* was used as a positive control template for amplifying gene encoding *Plasmodium 18s rRNA* and *MSP1*.
