# Supplementary material for: Human liver organoids are susceptible to Plasmodium vivax infection
Source: Malar J. 2024 Dec 5;23:368. doi: 10.1186/s12936-024-05202-8 (PMC11622667; doi:10.1186/s12936-024-05202-8)
Supplement: Supplementary file 3 — Additional file 3: Figure S3. Liver-stage infection of P. vivax in liver organoid. (A) Confocal microscopic observation of P. vivax UIS4, a protein located in parasitophorous vacuole membrane, in liver organoids. Under confocal images, nuclei were stained with DAPI (blue), while P. vivax UIS4 was detected using anti-PvUIS4 (green). Bright field (BF) images show the edge of the cell layer. Scale bar = 10 µm. [file 12936_2024_5202_MOESM3_ESM.pdf]

Additional file 3

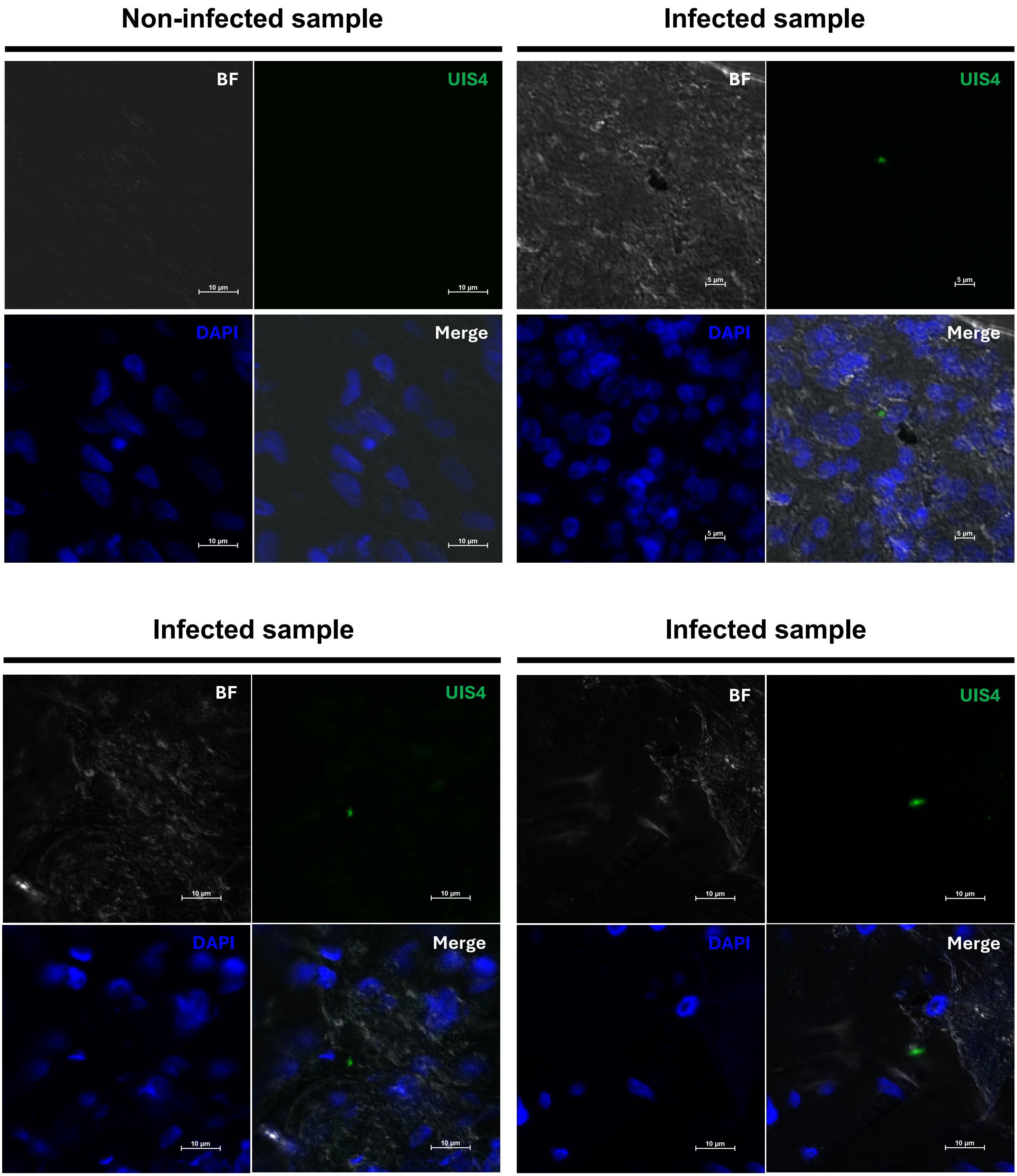

Figure S3. Liver-stage infection of *P. vivax* in liver organoid. Confocal microscopic observation of *P. vivax* UIS4, a protein located in parasitophorous vacuole membrane, in liver organoids. Under confocal images, nuclei were stained with DAPI (blue), while *P. vivax* UIS4 was detected using anti-*Pv*UIS4 (green). Bright field (BF) images show the edge of the cell layer. Scale bar = 10 µm.
