# Supplementary material for: Human liver organoids are susceptible to Plasmodium vivax infection
Source: Malar J. 2024 Dec 5;23:368. doi: 10.1186/s12936-024-05202-8 (PMC11622667; doi:10.1186/s12936-024-05202-8)
Supplement: Supplementary file 4 — Additional file 4: Figure S4. Protein‒protein interactions related to lipid and cholesterol metabolism in the liver. (A) Protein‒protein interaction (PPI) networks were constructed with Metascape. The different clusters are labeled with different colours. [file 12936_2024_5202_MOESM4_ESM.pdf]

Additional file 4

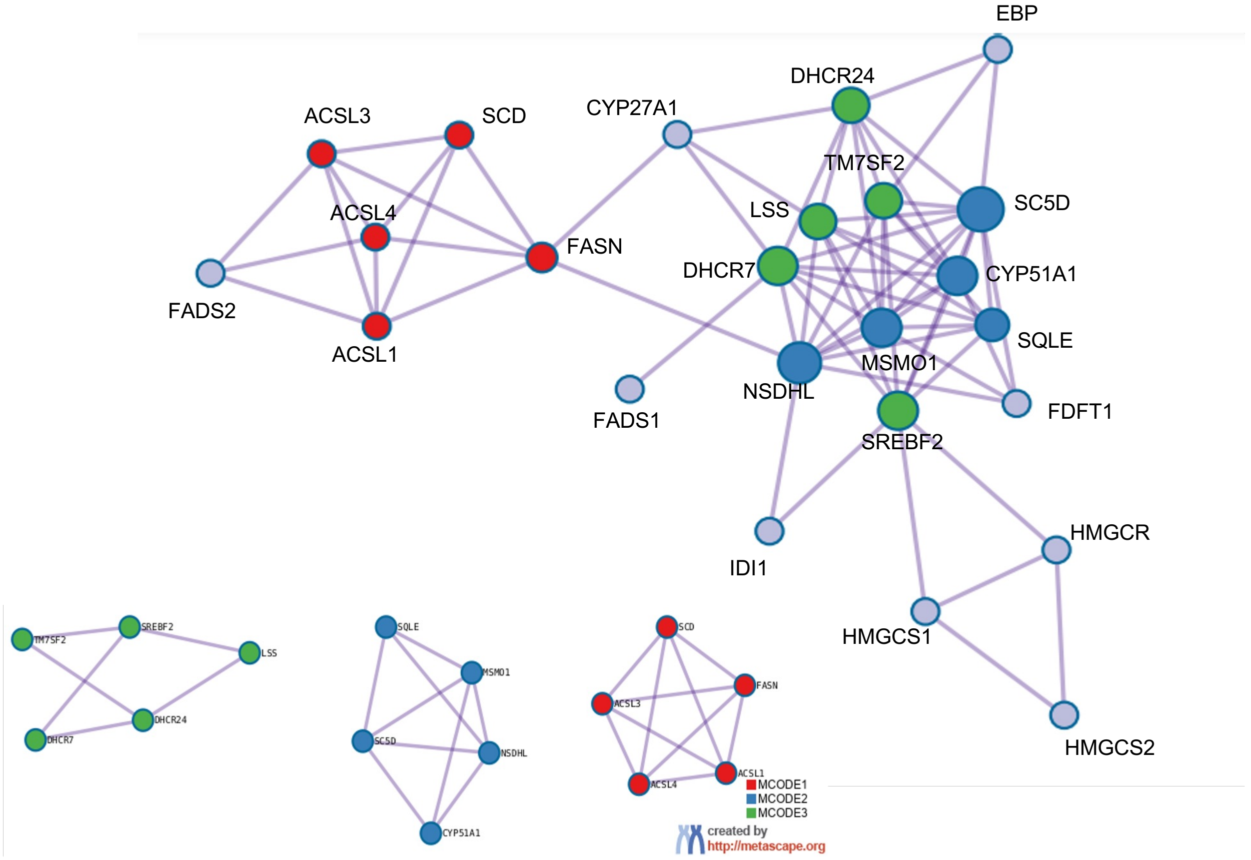

GO	Description	Log10(P)
WP4718	Cholesterol metabolism with Bloch and Kandutsch Russell pathways	-70.5
WP5329	Cholesterol biosynthesis pathway in hepatocytes	-47.9
R-HSA-191273	Cholesterol biosynthesis	-41.7

Color	MCODE	GO	Description	Log10(P)
(red)	MCODE_1	WP4724	Omega 9 fatty acid synthesis	-17.0
(red)	MCODE_1	WP357	Fatty acid biosynthesis	-15.9
(red)	MCODE_1	R-HSA-75105	Fatty acyl-CoA biosynthesis	-14.6
(blue)	MCODE_2	WP197	Cholesterol biosynthesis pathway	-16.8
(blue)	MCODE_2	hsa00100	Steroid biosynthesis	-16.1
(blue)	MCODE_2	R-HSA-191273	Cholesterol biosynthesis	-15.4
(green)	MCODE_3	R-HSA-191273	Cholesterol biosynthesis	-15.4
(green)	MCODE_3	WP4718	Cholesterol metabolism with Bloch and Kandutsch Russell pathways	-14.1
(green)	MCODE_3	hsa00100	Steroid biosynthesis	-12.2

Figure S4. Protein–protein interactions related to lipid and cholesterol metabolism in the liver. (A) Protein–protein interaction (PPI) networks were constructed with Metascape. The different clusters are labeled with different colors.
