# Supplementary material for: Human liver organoids are susceptible to Plasmodium vivax infection
Source: Malar J. 2024 Dec 5;23:368. doi: 10.1186/s12936-024-05202-8 (PMC11622667; doi:10.1186/s12936-024-05202-8)
Supplement: Supplementary file 5 — Additional file 5: Figure S5. Enrichment of human reticulocytes and surface marker identification. (A) Human reticulocytes after enrichment with 15% OptiPrep-KCl and staining with methylene blue dye. The reticulocytes presented precipitated blue RNA granules in the red cells (arrowhead). The percentage of reticulocytes was calculated under a light microscope for a minimum of 5,000 erythrocytes. (B) Dot plot of a representative flow cytometry analysis showing the reticulocytes before and after enrichment. The red blood cells (RBCs) were gated according to forward scatter height (FSC-H) and side scatter height (SSC-H). The reticulocytes were defined as CD71-positive and CD235a-positive cells. [file 12936_2024_5202_MOESM5_ESM.pdf]

Additional file 5

A

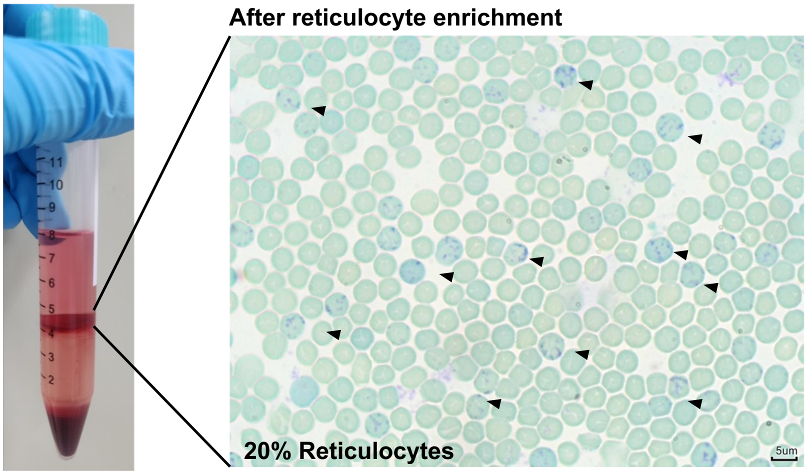

B

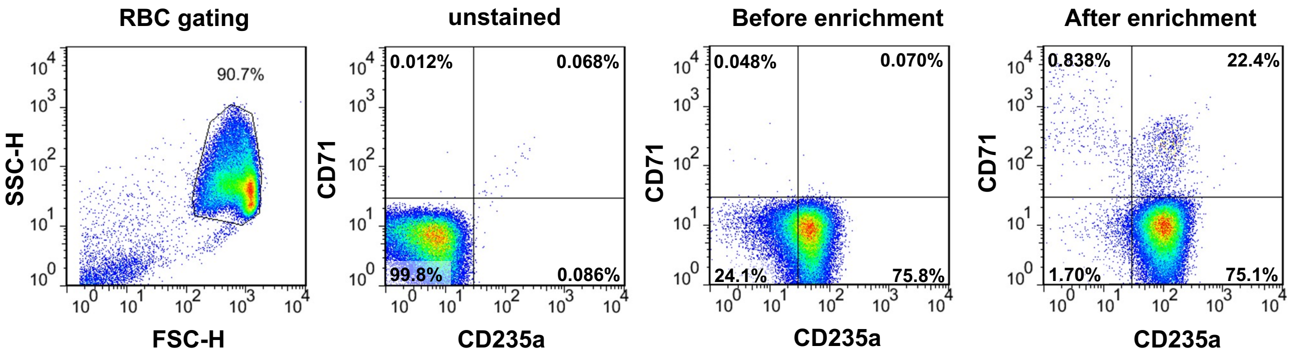

Figure S5. Enrichment of human reticulocytes and surface marker identification. (**A**) Human reticulocytes after enrichment with 15% OptiPrep-KCl and staining with methylene blue dye. The reticulocytes presented precipitated blue RNA granules in the red cells (arrowhead). The percentage of reticulocytes was calculated under a light microscope for a minimum of 5,000 erythrocytes. (**B**) Dot plot of a representative flow cytometry analysis showing the reticulocytes before and after enrichment. The red blood cells (RBCs) were gated according to forward scatter height (FSC-H) and side scatter height (SSC-H). The reticulocytes were defined as CD71-positive and CD235a-positive cells.
